# Single Inhaler LABA/LAMA for COPD

**DOI:** 10.3389/fphar.2019.00390

**Published:** 2019-04-25

**Authors:** Mario Malerba, Valentina Foci, Filippo Patrucco, Patrizia Pochetti, Matteo Nardin, Corrado Pelaia, Alessandro Radaeli

**Affiliations:** ^1^Respiratory Medicine, Department of Translational Medicine, University of Eastern Piedmont, Vercelli, Italy; ^2^Respiratory Unit, Sant’Andrea Hospital, Vercelli, Italy; ^3^Department of Medicine, Spedali Civili di Brescia, Brescia, Italy; ^4^Department of Medical and Surgical Sciences, Section of Respiratory Diseases, University “Magna Græcia” of Catanzaro, Catanzaro, Italy; ^5^Department of Emergency, Spedali Civili di Brescia, Brescia, Italy

**Keywords:** chronic obstructive pulmonary disease (COPD), LABA, LAMA, maintenance treatment, fixed dose combination (FDC)

## Abstract

Chronic obstructive pulmonary disease (COPD) is a common disabling disease characterized by progressive airflow obstruction. Great efforts were spent in the development of drugs able to improve symptoms, quality of life, reduce exacerbations, hospitalizations and the frequency of death of patients with COPD. The cornerstones of treatment are bronchodilator drugs of two different classes: beta agonists and muscarinic antagonists. Currently the Global initiative for COPD suggests the use of long acting beta agonists (LABAs) and long acting muscarinic antagonists (LAMAs) in combination for the majority of COPD patients, thus great interest is associated with the developing of LAMA/LABA fixed combination in the maintenance treatment of stable COPD. Many LAMA/LABA fixed dose combinations have been licensed in different countries and the clinical use of these drugs stimulated the performance of many clinical trials. The purpose of this review is a complete criticism of pharmacological and clinical aspects related to the use of LAMA/LABA single inhalers for the maintenance treatment of stable COPD, with particular mention to the most debated topics and future prospects in the field.

## Introduction

Chronic obstructive pulmonary disease (COPD) is believed to be the third leading cause of death worldwide by 2020 (WHO, 2008).

Chronic obstructive pulmonary diseases is a common disease characterized by respiratory symptoms and progressive airflow obstruction due to alveolar and bronchial abnormalities and inflammation caused by exposition to noxious substances (Global initiative for chronic obstructive Lung disease [GOLD], 2018). COPD is associated to dyspnea, cough, and sputum with lung hyperinflation. COPD is a disabling disease with huge impact on normal daily activities and limiting quality of life, the disease abruptly worsen due to exacerbations inducing a step down of health conditions, following which the recovery of breath function and activities is gradually slower and more difficult leading to disability and death (Global initiative for chronic obstructive Lung disease [GOLD], 2018).

Following this series of reasons there is a great impulse in the development of drugs able to improve symptoms, quality of life, reduce exacerbations, hospitalizations and the frequency of death of patients with COPD. The cornerstones of treatment are bronchodilator drugs of different classes including beta agonists and muscarinic antagonists (Montuschi and Ciabattoni, 2015).

Currently the Global initiative for COPD (GOLD) promotes the “ABCD” assessment of COPD patients based on symptoms severity (assessed by questionnaire) and exacerbation risk (low risk consisting in no more than one moderate – severe exacerbation during the past year). GOLD group A includes patients with low symptom severity and low exacerbation risk. GOLD group B includes patients with high symptom severity and low exacerbation risk. GOLD group C includes patients with low symptom severity but high exacerbation risk. GOLD group D includes patients presenting high symptom severity and high exacerbation risk (Global initiative for chronic obstructive Lung disease [GOLD], 2018).

Global initiative for COPD suggests the use of long acting beta agonists (LABA) and long acting muscarinic antagonists (LAMA) in combination for group B patients with persistent symptoms, group C patients with further exacerbations on LAMA treatment and for group D patients with or without the addition of inhaled corticosteroids (ICSs).

The administration of these two drugs simultaneously with the same device (single inhalator) should ensure a better adherence to the treatment and ad hoc dosages to produce a “synergistic effect between the two drugs respect to the administration of the two drugs separately (Tashkin and Ferguson, 2013).

Currently the LAMA/LABA combination in single inhaler device disposable on the market include Umeclidinium/ Vilanterol (Anoro^®^), Tiotropium/Olodaterol (Stiolto^®^), Glycopyrrolate/Formoterol (Bevespi^®^) and Glycopyrronium/Indacaterol (Ultibron^®^) available in the United States. Glycopyrronium/Indacaterol (Ultibro^®^), Tiotropium bromide/Olodaterol (Spiolto^®^), Umeclidinium/Vilanterol (Anoro^®^), and Aclidinium/Formoterol (Duaklir^®^ Genuair^®^, Brimica^®^ Genuair^®^) are available in the European Union (Table 1).

**Table 1:** LAMA/LABA fixed dose combinations licensed in different countries.

Drugs FDC	Dosage (μg)	Brand name	Device	Country
Umeclidinium	55	Anoro^®^	Ellipta (DPI)^®^	US and EU


Vilanterol	22			
Tiotropium	5	Spiolto^®^	Respimat (SMI)^®^	EU


Olodaterol	5	Stiolto^®^		US
Glycopyrronium	50	Ultibro^®^	Breezhaler (DPI)^®^	EU


Indacaterol	110			
Glycopyrronium	15,6	Ultibron^®^	Neohaler (DPI)^®^	US


Indacaterol	27,5			
Glycopyrronium	9	Brevespi^®^	Aerosphere (pMDI)^®^	US


Formoterol Fum.	4.8			
Aclidinium	340	Brimica^®^	Genuair (DPI)^®^	EU


Formoterol Fum.	12	Duaklir^®^		
Tiotripium	9 or 18	Duova^®^	DPI	Lebanon


Formoterol Fum.	6 or 12	Tioform^®^		India


*LAMA, long acting muscarinic antagonist; LABA, long acting beta agonist; FDC, fixed doses combination; DPI, dry powder inhaler; US, United States; EU, European Union; SMI, soft mist inhaler; pMDI, pressurized metered dose inhaler; Fum., fumarate.*

Purpose of this review is providing a complete criticism of LAMA/LABA single inhaler treatment in COPD with particular mention to the most debated topics and future prospects in the field.

## Pharmacology Mechanism of Action and Rationale for LAMA/LABA Fixed Dose Combinations in a Single Inhaler for COPD

**FIGURE 1:**
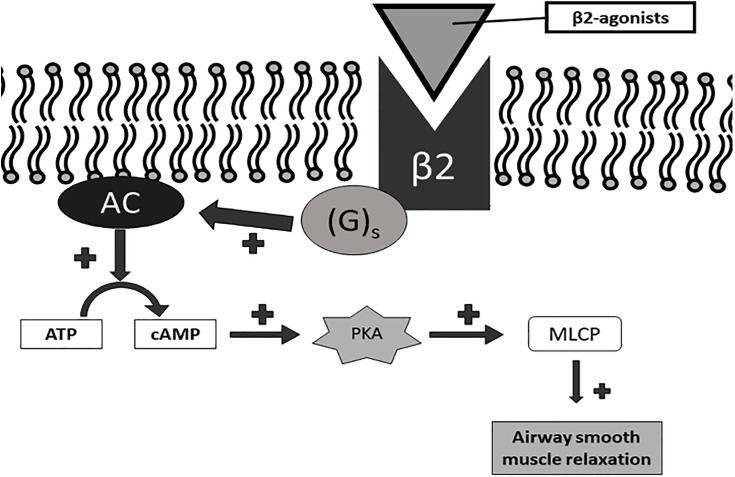
Parasympathic pathway involved in control of airway smooth muscle contraction. ACh, acetylcholine; M1, M1-muscarinic receptor; M2, M2-muscarinic receptor; M3, M3-muscarinic receptor.

Long acting beta agonist and LAMA are two major classes of bronchodilators and currently the principal medications for patients with COPD. LABA relax airway smooth muscle by linking with the beta2-adrenergic receptors. This linkage cause bronchodilation by inducing conformational changes in the post-synaptic β2 receptor on airway smooth muscle cells with consequent activation of a stimulatory guanosine-threephosphate-(GTP) binding protein (G2), increased adenylyl cyclase activity and cyclic adenosine monophosphate (cAMP) synthesis, Protein Kinase A activation and sequential intracellular events, including inhibition of myosin light chain kinase (MLCK), leading to reduction in smooth muscle airway contractility. This relaxation is also caused by activation of large conductance Ca^2+^ activated K^+^ channels via G2 which leads to plasma membrane hyperpolarization (Montuschi et al., 2016) (Figure 1).

**FIGURE 2:**
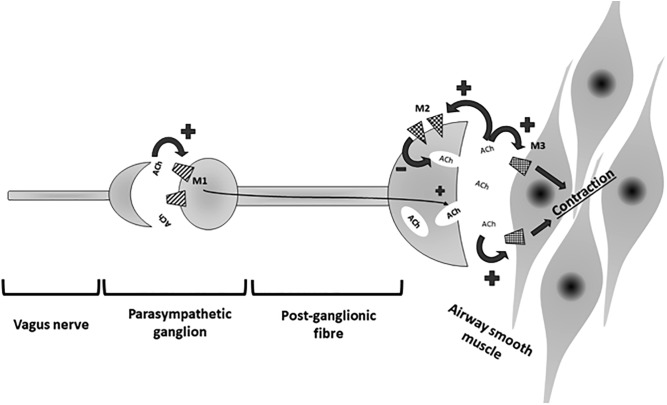
Signaling pathway of β2 receptor activation. Gs, Gs-protein; AC, adenylate cyclase; ATP, adenosine triphosphate; cAMP, cyclic AMP; PKA, protein kinase A; MLCP, myosin light chain phosphatase.

Long acting beta agonist can be divided into once-daily and twice-daily LABA. Once-daily LABA are currently called ultra-LABA. Ultra-LABA are ultra-long acting and are dosed once a day such as indacaterol (IND), olodaterol (OLO), and vilanterol (VIL); they provides both the quick bronchodilation effect similar to short-acting beta agonists, and a 24-h bronchodilation effect permitting the once-daily administration (Horita et al., 2017). Twice-daily LABAs are formoterol fumarate (FF) or propionate (FP) and salmeterol (SAL). LAMA block the bronchoconstrinction effect of acetylcholine on M3 muscarinic receptors expressed in airway smooth muscle; they have prolonged binding to M3 muscarinic receptors with faster dissociation from M2 muscarinic receptors (Global initiative for chronic obstructive Lung disease [GOLD], 2018). M3 receptors are coupled (GTP) – binding protein (G2) which regulates intracellular calcium concentration and calcium-modulated proteins (Pera and Penn, 2014) (Figure 2).

Long acting muscarinic antagonists such as tiotropium (TIO), umeclidinium (UMEC), and glycopyrronium (GLY) are long acting drugs and are dosed once a day, while aclidinium (ACL) is dosed twice a day.

**FIGURE 3:**
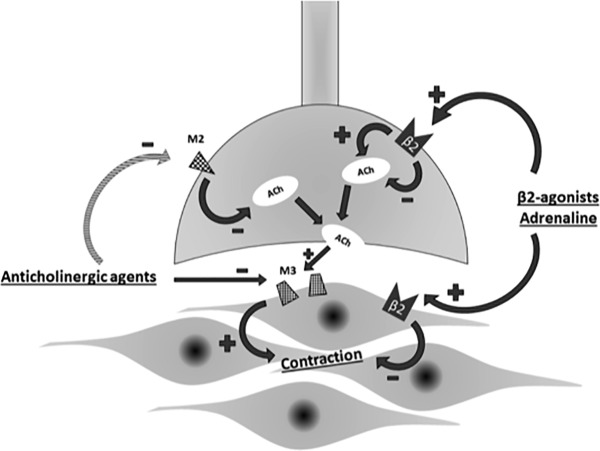
Interplay of adrenergic and cholinergic pathway on airway smooth muscle. ACh, acetylcholine; M2, M2-muscarinic receptor; M3,M3-muscarinic receptor.

Bronchodilator monotherapy is not always satisfactory for patients with advanced COPD. In that situation, a dual-bronchodilator therapy consisting of LAMA and LABA is a good option. LAMA/LABA fixed dose combinations (FDCs) have been shown to improve lung function, lung hyperinflation, exercise capacity, quality of life and exacerbation frequency thereby slowing disease progression in COPD (Global initiative for chronic obstructive Lung disease [GOLD], 2018). This combinations have a synergistic effect rather than just being additive one (Tashkin and Ferguson, 2013). Synergy is defined as the drug combination having a greater effect than would be expected from the mono-components alone. Synergy would be beneficial as a greater degree of bronchodilation could potentially be achieved at lower doses of the individual components thus minimizing side effects. LABAs activate pre-junctional β2-adrenoceptors and reduce acetylcholine release thereby prevent any functional competition by acetylcholine at post-junctional muscarinic receptors in the airways occupied by LAMAs. Thus LAMA/LABA combinations exploit both the adrenergic and cholinergic pathways in the airway smooth muscle to maximize bronchodilation (Lal and Strange, 2017) (Figure 3).

## LAMA/LABA Fixed Dose Combinations in a Single Inhaler for COPD: Evidences From Clinical Trials

LAMA/LABA combinations in a single inhaler with fixed dose are numerous. Here we resemble the principle international studies on efficacy and safety of LAMA/LABA combinations.

The main published studies on LAMA/LABA single inhaler treatments are summarized in Table 2.

**Table 2:** Main published studies on LABA/LAMA single inhaler for COPD.

Drugs (reference)	Competitor	Duration (weeks)	ITT population	Main results
UMEC/VIL 62.5/25 μg OD (Maleki-Yazdi et al., 2014)	TIO 18 μg OD	24	1191	Improv. t-FEV_1_ and HRQL vs. TIO
UMEC/VIL 62.5/25 μg OD (Donohue et al., 2015)	SAL/FP 250/50 μg TD	12	697	Improv. 0–24 h wmFEV_1_ and t-FEV_1_ vs. SAL/FP
TIO/OLO 5/5 μg OD (Derom et al., 2016)	TIO (various dosages)	52	11 *trials*	Improv. Pulm Funct. and HRQL vs. PLAC
	OLO (various dosages)			
	PLAC			
ENERGITO study	SAL/FP 250/50 μg TD	6	229	Improv. FEV_1_ AUC 0–12 and 0–24 vs. both competitors
TIO/OLO 5/5 μg OD	SAL/FP 500/50 μg TD			
TIO/OLO 5/2.5 μg OD (Beeh et al., 2016)				
SHINE study	IND 110 μg, GLY	26	2144	Improv. t- FEV_1_ vs. monocomponents, TIO and PLAC
QVA149 OD (Bateman et al., 2013)	150 μg			
	TIO 18 μg, PLAC			
	All OD			
SPARK study	GLY 50 μg OD	64	2224	Reduced mod to severe exacerbations rate vs. both
QVA149 OD (Wedzicha et al., 2013)	TIO 18 μg OD			Impov. t- FEV_1_ vs. both
FLAME study	SAL/FP 50/500 μg TD	52	3362	Reduced all severity exacerbation rate vs. competitor (reduced incidence of pneumonia vs. competitor)
QVA149 OD (Wedzicha et al., 2016)				
ILLUMINATE study	SAL/FP 50/500 μg TD	26	523	Improv. FEV_1_ AUC 0–12 vs. competitor
QVA149 OD (Vogelmeier et al., 2013)				
LANTERN study	SAL/FP 50/500 μg TD	26	744	Improv. t-FEV_1_ vs. competitor (reduced incidence of pneumonia vs. competitor)
QVA149 OD (Zhong et al., 2015)				
FLIGHT 1 and 2 and 3 study	GLY 15.6 μg TD	12	2038	Improv. FEV_1_ AUC 0–12 vs. monocomponents and PLAC
GLY/IND 15.6/27.5 μg TD (Mahler et al., 2015)	IND 27.5 μg TD			
	PLAC			
PINNACLE 1 and 2 study	GLY 18 μg TD	24	3274	Improv. t-FEV_1_ vs. competitors
GLY/FF 18/9.6 μg TD (Martinez et al., 2017b)	FF 9.6 μg TD			
	TIO 18 μg OD (PINNACLE 1)			
	PLAC			
PINNACLE 3	GLY 18 μg TD	26	892	Improv. tFEV_1_, RMU, TDI vs. monocomponents and TIO
GLY/FF 18/9.6 μg TD (Hanania et al., 2017)	FF 9.6 μg TD			
	TIO 18 μg OD			
ACLIFORM study	ACL 400 μg TD	24	1729	Both Improv. 1-h post-dose FEV_1_ vs. ACL 400 μg
ACL/FF 400/12 μg TD	FF 12 μg TD			Both Improv. t-FEV_1_ vs. FF 12 μg
ACL/FF 400/6 μg TD (Singh et al., 2014)	PLAC			Both Improv. TDI vs. PLAC
AUGMENT study	ACL 400 μg TD	24	1692	Both Improv. 1-h post-dose FEV_1_ vs. ACL 400 μg
ACL/FF 400/12 μg TD	FF 12 μg TD			ACL/FF 400/12 Improv. t-FEV_1_ vs. FF 12 μg
ACL/FF 400/6 μg TD (D’Urzo et al., 2014)	PLAC			ACL/FF 400/12 Improv. TDI and HRQL vs. PLAC
TIO/FF 18/12 μg (Salvi et al., 2014)	TIO 18 μg	Single dose	44	Faster bronchodilator response (FEV_1_, FVC) vs. TIO
				Improv. mean maximum change in FEV1, FVC vs. TIO
				Improv. FEV_1_, FVC and IC AUC (0–24 h) vs. TIO	


*UMEC, umeclidinium; VIL, vilanterol; OD, once daily; TIO, tiotropium; t-FEV_*1*,_ through forced expiratory volume at the first second; HRQL, health-related QVAlity of life; Vs, versus; SAL, salbutamol; FP, fluticasone propionate; TD, twice daily; wm, weighted mean; OLO, olodaterol; PLAC, placebo; AUC, area under curve; QVA149, glycopyrronium 50 μg + indacaterol 110 μg; GLY, glycopyrronium; IND, indacaterol; FF, fluticasone fumarate; TDI, transitional dyspnea index; RMU, rescue medication use; ACL, aclidinium; FVC, forced vital capacity; IC, inspiratory capacity.*

### Umeclidinium/Vilanterol

Numerous international clinical trials have evaluated the efficacy and safety of UMEC/VIL against placebo, monocomponents, TIO and associations between LABA and ICS (Malerba et al., 2016). We summarize the principal studies: a 24 weeks trial UMEC/VIL 125/25 μg once daily (OD) was tested vs. UMEC 125 μg, VIL 25 μg and placebo (OD) in more than 1000 COPD patients. Combined treatment was superior to placebo and monocomponents in improving difference from baseline of through forced expiratory volume at the first second (tFEV_1_), transitional dyspnea index (TDI) and rescue medication use (RMu) (Celli et al., 2014).

Decramer and colleagues conducted two 24 weeks trial in more than 800 COPD patients (each study) testing UMEC/VIL 125/25 μg, UMEC/VIL 62.5/25 μg, TIO 18 μg, VIL 25 μg or UMEC 125 μg (all OD). They reported that both associations treatments were superior to TIO, UMEC, and VIL alone in improving tFEV_1_ and RMu (Decramer et al., 2014). Another (Maleki-Yazdi et al., 2014) 24-week, multicenter, randomized, blinded, double-dummy, parallel-group study Phase III study compared UMEC/VIL 62.5/25 μg, to TIO 18 μg OD. Significant improvements in trough FEV_1_ was observed for UMEC/VIL versus TIO. Also, UMEC/VIL improved health-related quality of life (HRQL), and reduced the requirement for RMu compared with TIO.

Three 12-week trials enrolling more than 2000 patients with COPD compared UMEC/VIL 62.5/25 μg OD with Salmenterol/Fluticasone propionate (SAL/FP) 250/50 μg twice daily (TD) and 500/50 μg TD (Donohue et al., 2015; Singh et al., 2015). In these studies UMEC/VIL provided an improved of pulmonary function (mean change from baseline in tFEV_1_) in all trials. Only one study reported a reduction in RMu in favor of patients treated with UMEC/VIL (Donohue et al., 2015).

In summary the considered trials concluded that once-daily UMEC/VIL 62.5/25 μg OD, was well-tolerated, provided clinically-significant improvements in lung function and symptoms in COPD patients.

UMEC/VIL 55–22 μg is commercialized with the name of Anoro^®^ and delivered by the Ellipta^®^ dry powder inhaler (DPI).

### Tiotropium/Olodaterol

The efficacy and safety of this combination was studied several randomized, double-blind, parallel-group, multicenter trials (Derom et al., 2016). In these studies, over 10,000 patients have been involved, for simplicity we consider only the dosage of 5/5 μg OD that is recommended and available on market. The majority of these studies involved patients with COPD GOLD II and III stage.

TIO/OLO 5/5 μg not only improved pulmonary function more than placebo but also resulted in statistically significant improvements on dyspnea, RMu, HRQL, and exercise endurance.

TIO/OLO 5/5 μg differed significantly from TIO 5 μg monotherapy in terms of pulmonary function [FEV_1_ area under curve (AUC)0–24, FEV_1_ AUC0–12, and FEV_1_ AUC12–24] and reducing symptoms of dyspnea (TDI and HRQ). Comparison between TIO/OLO 5/5 μg FDC OD and OLO 5 μg OD, was generally statistically but not clinically significant in favor of fixed dose association not reaching the minimal clinical important difference (MCID). In a 6-week trial that enrolled more than 200 patients with COPD, TIO/OLO 5/5 μg was significantly more effective than SAL/FP TD at improving pulmonary function (ENERGITO study) (Beeh et al., 2016).

TIO/OLO 5/5 mg is formulated in the Respimat^®^soft-mist inhaler (SMI) commercialized under the name of Spiolto^®^(EU) or Stiolto^®^(US).

### Glycopyronium/Indacaterol

The fixed-dose combination of LAMA GLY 50 μg plus LABA IND 110 μg (QVA149) has been shown in a series of clinical trials to be more effective than placebo and the single components with regard to lung function, symptoms, and quality of life outcomes, being as safe as the single components and placebo. All the considered studies were randomized double-blind.

SHINE study (Bateman et al., 2013) is a multicenter, 26-week trial which evaluated the safety and efficacy in terms of tFEV 1 of QVA149 in comparison to IND, GLY, TIO, and placebo (OD) in patients with moderate-to-severe COPD. Trough FEV_1_ at week 26 was significantly improved with QVA149 compared to all other treatment arms, with a safety and tolerability profile similar to placebo.

SPARK (Wedzicha et al., 2013) is a 64-week, parallel-group, active controlled study to evaluated the effect of QVA149 vs. GLY 50 μg and TIO 18 μg (OD) in 2224 patients with severe-to-very severe COPD (GOLD COPD stages III and IV and one or more moderate COPD exacerbation in the past year). QVA149 was found to significantly reduce the rate of moderate to severe COPD exacerbations by 12% compared to GLY, and by 10% compared to TIO; however, with not significant differences. QVA149 also significantly improved tFEV 1 and quality of life as compared to GLY and TIO without any increase in adverse events.

FLAME A 52-week multi-center, parallel-group, active controlled study to compare the effect of QVA149 OD with SAL/FP TD on the rate of exacerbations in 3332 patients with moderate-to-very severe COPD. QVA149 reduced the annual rate of all COPD exacerbations (the rate was 11% lower in the QVA149 group than in the SAL/FP group) (Wedzicha et al., 2016).

ILLUMINATE study (Vogelmeier et al., 2013) is a 26 weeks parallel-group study, in which 523 patients with moderate to severe COPD were assigned to OD QVA149 or TD SAL/FP 500/50 μg. At week 26 FEV_1_ AUC 0–12 h was significantly higher with QVA149 than with SAL/FP, with a similar incidence of serious adverse events.

LANTERN is a 26-week parallel-group study conducted to assess the efficacy and safety of OD QVA149 compared to TD SAL/FP 500/50 μg in 744 patients with moderate-to-severe stable COPD with or without exacerbations in the previous year. QVA149 had significant superiority to SAL/FP for tFEV_1_ and for the standardized AUC from 0 to 4 h. QVA149 showed similar improvements in TDI focal score, St George Respiratory Questionnaire total score, and RMu compared to the other active arm. However, QVA149 significantly reduced the rate of moderate or severe exacerbations by 31% (*p* = 0.048) over SAL/FP. The incidence of pneumonia was threefold lower with QVA149 (0.8%) (Zhong et al., 2015).

The FDA approved dose for GLY/IND in the US is 15.6/27.5 μg TD based on the FLIGHT1 and FLIGHT2 studies (Mahler et al., 2015) while in the European Union the approved dose for GLY/IND is 50/110 μg OD. Studies have shown that the two formulations have similar effects. The EXPEDITION program consisted of FLIGHT 1, 2, and 3 studies which tested GLY/IND 15.6/27.5 μg TD in US. It has shown a significant improvement in lung function for the FDC compared to monocomponents and in HRQL and RMu compared to placebo (Mahler et al., 2015).

GLY/IND association 50–110 μg is marketed in EU with the brand name of Ultibro^®^, delivered by Neohaler^®^DPI.

GLY/IND association 15,6–27,5 μg is marketed in United States with the brand name of Ultibron^®^, delivered by the Breezhaler^®^DPI.

### Glycopyrrolate/Formoterol

Two 24-weeks, randomized, double blind, and placebo controlled phase III trials: PINNACLE-1 and PINNACLE-2 assessed the clinical efficacy and safety of GLY/FF 18/9.6 μg fixed dose association in patients with moderate and very severe COPD (Martinez et al., 2017b). Patients received GLY/FF, GLY 18 μg, FF 9.6 μg, or placebo (all TD), or TIO 18 μg. At the end of the trials patients were randomized to prosecute the trial for 52 w: PINNACLE-3 (Hanania et al., 2017). At week 24, significant differences in change from baseline in the trough FEV_1_ for GLY/FF vs. placebo, GP, and FF were seen in both PINNACLE-1 and PINNACLE-2. The incidence of adverse events was similar between all treatment arms. Improvements in efficacy endpoints were also sustained over 52 weeks.

GLY/FF is delivered by a Metered Dose Inhaler Formulated Using Co-Suspension Delivery Technology and commercialized with the name of Bevespi^®^ (available only in US market).

### Aclidinium/Formoterol

Two 6-month, multicentre, randomized studies (ACLIFORM and AUGMENT) have evaluated the safety and efficacy of ACL/FF FDC. The first study (Singh et al., 2014) compared the 400/6 μg and 400/12 μg combinations of ACL/FF with ACL 400 μg, FF 12 μg and placebo (all TD). Both combinations demonstrated statistically significant improvements to placebo in from baseline in 1-h post-dose FEV_1_, tFEV_1_ in the changes in TDI. At week 24, both combinations showed significant improvements from baseline in 1-h post-dose FEV_1_ versus ACL and tFEV_1_ versus FF.

AUGMENT study (D’Urzo et al., 2014) was conducted in 1692 stable COPD patients comparing ACL/FF 400/12 μg, ACL/FF 400/6 μg, ACL 400 μg, FF 12 μg, or placebo (all TD).

Statistically significant improvements at week 24 were observed in the co-primary endpoints of FEV_1_ 1-h post-dose, by the 400/12 μg combination of ACL/FF, versus 400 μg ACL and for tFEV_1_ versus FF 12 μg. The 400/6 μg combination produced statistically significant improvements in (FEV_1_) 1-h post-dose versus ACL, but for the change from baseline tFEV_1_ did not reach significance as compared to FF 12 μg. The 400/12 μg combination induced a Peak FEV_1_ improvement at 24 weeks by 285 ml versus placebo (*p* < 0.0001) and the 400/6 μg combination by 259 ml with versus placebo (*p* < 0.0001).

A pooled analysis of these two trials (Bateman et al., 2015) revealed that 400/12 combination improved TDI focal score, HQRL, overall night-time and early-morning symptom severity versus placebo and both monotherapies at Week 24 (all *p* < 0.05).

The rate of moderate or severe exacerbations was significantly reduced with ACL/FF 400/12 μg compared with placebo (*p* < 0.05) but not monotherapies. 400/12 μg ACL/FF combination was the most effective dose and was found well-tolerated and safe.

ACL/FF 400/12 is commercialized with the names of Duaklir^®^ and Brimica^®^, delivered by the Genuair^®^ dry powder inhaler (available only in European market).

### Tiotropium/Formoterol

A single study enrolled 44 COPD subjects in a randomized, double-blind, multi-center, cross-over study. On two separate days 18 μg TIO and 18 μg TIO plus 12 μg FF (single dose) were administered via pressurized metered dose inhalers. The results showed that the combination of TIO plus FF showed a faster onset of bronchodilator response (*p* < 0.01 for FEV_1_ and forced vital capacity FVC), a greater mean maximum change in FEV_1_ (*p* = 0.01) and FVC (*p* = 0.008) and greater AUC0–24 h values for FEV_1_, FVC and inspiratory capacity (IC) compared with TIO alone. In the combination treated group Trough FEV_1_ and FVC values were also improved (Salvi et al., 2014).

TIO/FF is commercialized only in few countries with the name of DUOVA^®^ (Lebanon) with different dosages (9/6 mcg; 9/12 mcg and 18/12 mcg) and TIOFORM^®^ (India) 18/12 mcg; 9/6 mcg.

## The Role of LAMA/LABA Fixed Dose Combinations in a Single Inhaler for COPD Therapy in Global Initiative for Obstructive Lung Disease Recommendations

The rationale for fixed combination bronchodilator therapy in COPD is based on the increased bronchodilation and reduced side-effects compared to the single bronchodilators effects (Cazzola and Molimard, 2010). Since 2011, and after their last revision, GOLD recommendations introduced ABCD assessment tool to classify patients in four groups depending on symptoms and their history of exacerbations. Together with clinical and functional evaluation, ABCD assessment is crucial for stratification of prognosis and to decide which is the better therapy for patients (Vanfleteren et al., 2013; Soriano et al., 2015). In the management of stable COPD patients, the identification and reduction of exposure to risk factors are fundamental. The aim of pharmacological treatment is to reduce symptoms, the risk and severity of exacerbations in addition to improve health status and tolerance to exercise (Global initiative for chronic obstructive Lung disease [GOLD], 2018). Therapeutic decisions are strictly dependent to availability of medications and patient’s response or preference; nevertheless, since drugs are delivered with inhalators, a proper inhaler technique is required to take correctly medicaments.

Global initiative for COPD recommendations provide a model for initiation of the treatment and for subsequent adjustments such as escalation and/or de-escalation according to following evaluation of symptoms control and risk of exacerbations. The combined therapy with LABA and LAMA plays an important role in therapeutic strategies three out of four ABCD groups:

**Group B:** in group B, characterized by patients with high symptoms burden but low number of moderate/severe exacerbations, the initial therapy should be based on a long acting bronchodilator, LABA or LAMA (Barr et al., 2005; Appleton et al., 2006). In this group of patients, when there is the persistence of dyspnoea despite a monotherapy with a bronchodilator, it’s recommended to add a second bronchodilator (Karner and Cates, 2012). This approach originates from the results of a meta-analysis that included four studies (Aaron et al., 2007; Vogelmeier et al., 2008; Tashkin et al., 2009; Mahler et al., 2012).

Aaron et al. (2007) investigated SAL and TIO, Vogelmeier FF and TIO; both studies demonstrated a significant reduction of St George’s Respiratory Questionnaire (SGRQ) score, therefore an improvement of quality of life, for the group of patients in treatment with combined therapy LABA and TIO. Same studies did not report a reduction of hospital admission for any cause, neither for exacerbations. Taking into account the lung function, all the studies included in the meta-analysis demonstrated an improvement in pre-bronchodilator FEV_1_ at the end of the study for the LABA + TIO groups.

Recently Calzetta et al. (2016) published a review and meta-analysis on duration of treatment of LABA and LAMA combinations in COPD patients; they included 14 studies that reported results of 20 randomized controlled trials. In these studies the drugs used for LAMA/LABA therapy were: GLY/IND, UMEC/VIL, TIO/OLO, ACL/FF with different regimens of administration (once or twice daily). LAMA/LABA significantly improved trough FEV_1_, SGRQ and TDI after 3, 6, and 12 months of treatment, when compared with monocomponents. Again Rodrigo et al. (2017) in a recent meta analysis demonstrated the effectiveness of dual broncodilation respect monocomponents, in particular for changes of through FEV_1_ when compared to LABA. The stability of the effect of combined therapy on FEV_1_ lasts for 12 months and remain stable and superior for the whole period of observation.

For those patients in group B that complain severe breathless, GOLD recommendations suggest to start with combination therapy. This approach originates from a study conduced by Martinez et al. (2017a) that evaluated *post hoc* analysis of pooled data from PINNACLE-1 and PINNACLE-2 phase III studies, evaluating GLY/FF combination versus single components, on lung function, exacerbation and baseline symptom burden (measured with COPD Assessment Test, CAT) (Martinez et al., 2017a). The significant improvement of SGRQ score obtained by combination therapy respect to monotherapies was greater in those patients with baseline CAT score ≥ 20 points. Similar results have been obtained considering other clinical parameters such as function of baseline symptom burden.

The GOLD Guidelines authors recommended that if the addition of the second bronchodilator did not reduce symptoms, the treatment could be stepped down to a single bronchodilator (LABA or LAMA). In this case it’s mandatory to evaluate or re-evaluate comorbidities that could be responsible of the poor symptoms’ control and affect the patient’s prognosis (Lange et al., 2012; Agustí, 2013).

**Group C:** in group C, characterized by patients with low symptoms burden but high number of moderate/severe exacerbations, the initial therapy should be based on a single long acting bronchodilator, LABA or LAMA. The two studies that investigated this approach concluded that LAMA was superior to LABA in prevention of exacerbations and the GOLD authors recommend to start with a LAMA in this group of patients (Vogelmeier et al., 2011; Decramer et al., 2013).

In those patients with persistent exacerbations, the addition of a second long acting bronchodilator or a combination of a LABA and an ICS may be beneficial (Miravitlles et al., 2017). In SPARK study, the annual rates of moderate or severe exacerbations and all exacerbations were lower in the group of patients treated with GLY/IND combination versus GLY alone (Wedzicha et al., 2013).

Due to an increase of the risk of development of pneumonia in some patients related to ICS, the first choice of combination therapy is LAMA/LABA even if not resulting from studies complaining patients in group C (Zhong et al., 2015; Wedzicha et al., 2016).

**Group D:** in group D, characterized by patients with high symptoms burden and high number of moderate/severe exacerbations, the initial therapy should be based on a combination bronchodilator therapy LAMA/LABA. This assumption is mainly based on results of Wedzicha et al. (2013, 2016) previously mentioned studies on combined LAMA/LABA versus monotherapies and LAMA/LABA versus ICS/LABA. Same results have been obtained in DYNAGITO trial with TIO/OLO dual therapy versus TIO alone (Calverley et al., 2018). Patients belonging to group D could be naive patients (at first diagnosis), or could be shifted to this group coming from group B, in case of increased number of exacerbations during the previous year, or from group C, in case of increase of symptoms’ burden. Those patients in treatment with combined LAMA/LABA bronchodilation, could be stepped up to triple-therapy LAMA/LABA/ICS in case of further exacerbations.

## LAMA/LABA Single Inhaler: Devices

Bronchodilators consist in a complex and technological drug delivery system composed by a specific drug formulation and delivery device that made inhalable the drug inside. The devices are substantially divided in: active devices, where the energy needed to create the aerosol is generated by the same inhaler through a complex technological system, and passive devices where the aerosolization of the drug is dependent from the inhaled air stream.

Inhalation devices can be distinguished in three types: pressurized metered dose inhalers (pMDIs), dry powder inhalers (DPIs) and, recently, soft mist inhaler (SMI, Boehringer Ingelheim). A comparison among inhaler devices in LABA/LAMA single inhaler treatment is reported in Table 3.

**Table 3:** Principal differences between inhaler devices used to deliver LAMA/LABA FDC.

Inhalers	Drug delivered	Type	Characteristics
BREEZHALER^®^ (NEOHALER IN US)	GLY/IND	DPI	Must be loaded by patient
			Single dose
			Breath activated
GENUAIR^®^	ACL/FF	DPI	Pre-metered
			Multidoses
			Breath activated
			Acustic feedback
ELLIPTA^®^	UMEC/VIL	DPI	Pre-meterd
			Multidose
			Breath activated
RESPIMAT^®^	TIO/OLO	SOFT MIST	Nebulizer
			Dissipating a solution mechanically activated
AEROSPHERE^®^	GLY/FF	pMDI	Pressurized canister
			Multidose
			Manually activated


*LAMA, long acting muscarinic antagonist; LABAs, long acting beta agonists; GLY, glycopyrronium; IND, Indacaterol; DPI, dry powder inhaler; ACL, aclidinium; FF, formoterol fumarate; UMEC, umeclidinium; VIL, vilanterol; TIO, tiotropium; OLO, olodaterol; pMDI, pressurized metered dose inhaler.*

### Pressurized Metered Dose Inhalers

In pMDIs the active drugs are dissolved or suspended in a propellant, a mixture of propellants, or a mixture of solvents and they are delivered via a compact pressurized aerosol dispenser (canister) by pushing the canister down into the holder. The canister contains several 100s metered doses of the medication. PMDIs are therefore not breath-activated and require the user to coordinate pressing down the canister and inhaling the medication.

### Dry Powder Inhalers

Dry powder inhalers contain the drug in dry state; the devices are usually constituted by a powder formulation, a dose metering mechanism that contains or measures a single dose of the drug, a powder de-agglomeration principle and a mouthpiece (Frijlink and De Boer, 2004). The vast majority of DPIs are breath-actuated devices, using a passive mechanism, without the need of patient-device coordination; for a proper dose release the patient is required to make a minimal inspiratory effort.

The delivery of the formulation is consequent an active process usually initiated by an inhalation maneuvre of the patient; then the drug formulation is de-agglomerated or dispersed in an aerosol of small and inhalable particles of active agent and excipients. Each of these processes is dependent from several factors such as the powder formulation, the DPI used and patient’ inspiratory effort. Indeed, in DPIs the inhalation maneuver is crucial and reflects the deposition rate of active principle in the respiratory tract; each DPI device has a different inhalation maneuver and the ability to perform a correct process is dependent from patient’s characteristics such as age and clinical condition (Brocklebank et al., 2001; Lavorini et al., 2008; Pedersen et al., 2010; Inhaler Error Steering Committee et al., 2013; Lexmond et al., 2014).

DPIs up to now used in the delivery of LAMA/LABA drugs for COPD patients are:

*Breezhaler*^®^ (Novartis, A.G., Basel, Switzerland) is a single-dose, breath-actuated DPI that releases a dry powder contained a pierced gelatin capsule loaded in the device by the patient before the inhalation. Brochodilators combination used is GLY/IND.

*Genuair*^®^ (AstraZeneca, Cambridge, United Kingdom) is a pre-metered multidose, breath-actuated, medium-resistance DPI; it provides both visual and acoustic feedback to indicate the correct inhalation of the dose. Bronchodilators combination used is ACLI/FF (van der Palen et al., 2013).

*Ellipta*^®^ (GlaxoSmithKline, Brentford, United Kingdom) is a pre-metered multidose, breath-actuated DPI; bronchodilators combinations used is UMEC/VIL (Komase et al., 2014).

Some studies investigated satisfaction, preference, and error occurrence of these DPIs. The preference of the patients and his skills in using the inhaler device can affect the effectiveness of the treatment. The ease of use is one of the most important characteristics the a device must have, in particular for elderly because they represent a huge part of patients affected by COPD (Man et al., 2018). The recent study conducted by Man et al. (2018) compared these three devices and evaluated satisfaction, error occurrence during use of DPIs (evaluating critical and non-critical errors). Authors demonstrated that Breezhaler^®^ had the highest score for “comfort”; this may be related to the shape, size of the device in particular for female population, but Breezhaler^®^ had a significantly lower score for “ease of dose preparation.” When authors investigated the “clarity to indicate correct dose preparation” and “clarity of indicate correct inhalation” Genuair^®^ received higher scores and this is due to the devices feedback mechanism that informs the patient to the correct preparation and inhalation of the loaded dose. Indeed, adherence to therapy is strictly related to the patient’s confidence of regular inhalation of the drug (Rajan and Gogtay, 2014). Satisfaction varies with the age of patients; younger patients gave higher scores to Ellipta^®^ than Breezhaler^®^ for “ease of operation” and “handling time,” and this results suggest that handling procedures of Ellipta^®^could be difficult for the older population (Svedsater et al., 2013; Komase et al., 2014).

### Comparing pMDIs and DPIs

Pressurized metered dose inhaler and DPIs have their advantages and disadvantages. PMDIs require the user to coordinate pressing down the canister and inhaling the medication while DPIs are activated by breath; however, the inspiratory flow rate is a disadvantage of DPIs. The rate required to deliver the medication in pMDI inhalers is low (about 30 L/min) while the rate required for DPIs is higher (differs based on the build of the inhaler). This would make it more difficult for some patients to be able to deliver the medication properly. DPIs also require the patient not to disperse medication via exhalation into the device prior to using. Another disadvantage of pMDIs is the propellant; previously chlorofluorocarbon (CFC) was used now substituted by hydrofluoroalkane (HFA) and this can cause the inhaler to be more expensive.

### Soft Mist Inhaler

Soft mist inhaler Respimat^®^ is one of the more recently inhalers available; it’s a nebuliser dissipating a solution composed by fine droplets of active agents. SMI is different from traditional nebuliser since it is portable and doesn’t require a power source to work because it’s activated mechanically. As pMDIs, the formation of the aerosol is instantaneous but for a correct inhalation it’s required a higher coordination (Lavorini, 2013). Nevertheless, even if SMI takes a longer time to generate an entire aerosol, the nebulization is emitted in a slow-moving mist fashion, allowing a higher lung deposition (Dalby et al., 2004). Due to the combination of smaller particle size, lower velocity and longer duration of the nebulization, a smaller dose of bronchodilators ensure the same level of efficacy and safety than metered dose inhalers. An higher lung deposition of nebulized drugs with SMI was demonstrated with a radio labeled drug particles (Newman et al., 1996, 1998; Steed et al., 1997; Pitcairn et al., 2005; Anderson, 2006).

LAMA/LABA combination administered with Respimat^®^ is TIO/OLO Dal Negro and Povero (2016) compared some devices in terms of patients’ preference and acceptability; Respimat^®^ and Genuair^®^ were preferred to Breezhaler^®^ reporting less difficulties in understanding maneouvers for activate and correctly practicing the inhalation. Respimat^®^ has been judged the easiest to use, the less problematic and the most easy learned device at first attempt of use (Dal Negro and Povero, 2016).

## Critical Issues for LABA/LAMA Combinations in a Single Inhaler Therapy for COPD

Dual bronchodilation treatment with LAMA/LABA FDC has demonstrated to be able to improve quality of life in COPD patients in terms of symptoms scores, rescue medication use (van Noord et al., 2005) and lung hyperinflation when compared to placebo. In comparison to in several trials (Bateman et al., 2013; Celli et al., 2014; Decramer et al., 2014; Mahler et al., 2015; Derom et al., 2016; Martinez et al., 2017b) as exercise tolerance (Berton et al., 2010). Moreover LAMA/LABA combinations have been noted to be superior compared to LABA/ICS combined treatment for COPD with at least one exacerbation in the previous year (Wedzicha et al., 2016). A meta-analysis of heterogeneous studies comparing LAMA/LABA with LABA/ICS (Horita et al., 2017) concluded that LAMA/LABA has fewer exacerbations, a larger improvement of FEV_1_, a lower risk of pneumonia, and more frequent improvement in quality of life indicators.

Clearly none of the pharmacological treatments have ever shown a significant impact on COPD mortality, however we believe that the effects of the LABA/LAMA combination treatment observed improving patient quality of life represent main issues in the treatment of COPD.

### Trials Limits

Concerns have been raised about the ability of large trials to actually identify the potential benefits of the investigated treatments. In particular, classical trials focused on the benefits of lung function (FEV_1_ changes in particular) as a primary endpoint and this could lead to an underestimation of the impact of the treatments studied on the improvement of endurance symptoms, frequency of exacerbations and hospitalizations and quality of life in general. It has also emerged how focusing on the change in FEV_1_ is deeply influenced by the choice of patients enrolled and may not translate into real life in an equally effective treatment, or vice versa fails to highlight an effective clinical impact in patients who, however, qualitatively improve quality of life performance. For these reasons clinical trials aimed at the ability to detect different endpoints capturing different aspects of COPD pathobiology such as real life clinical improvement are claimed. It is important to underline that the results of the different meta-analysis reported need to be taken with caution because of differences in the studied population and in the outcomes. This heterogeneity may result in difficulties to translate results in real life. Moreover LAMA/LABA direct comparison in head-to-head trials would be suggested.

#### FDCs Are Better Than Monocomponent Treatment

The administration of two drugs simultaneously with the same device should ensure a better adherence to the treatment (Tashkin and Ferguson, 2013) Using together two bronchodilators with different mechanism of action can overcome specific patient dissimilarities in the response to different treatments (Cazzola and Molimard, 2010), which may be due to different distribution of different receptors (beta agonists and muscarinic) in the lungs (Ikeda et al., 2012; Cazzola et al., 2015). However, the impossibility of change in the individual dosages of the two components may lead to potential problems in relation to individual side effects to one of the components. In the clinical trials these problems have never been observed and all LAMA/LABA FDC have shown optimal tolerability profiles. However, only the observation in real life clinical setting can reassure us about this aspect. Until now, the experiences reported in the daily clinical practice have given encouraging results, but we should keep the guard high in the detection of possible tolerance problems, especially in complex patients with multiple associated diseases usually excluded from clinical trials.

### Dosing and Administration

It is necessary to underline that the various LAMA/LABA combinations available on the market are provided in a single fixed dose regimen, which allows poor treatment flexibility and the impossibility of administering different doses to different patients (e.g., in the presence of relevant differences in BMI in patients routinely excluded from large clinical trials).

Some other aspects may generate uncertainties in the interpretation of data received from trials and in the prescription or dispensation of the drug: some formulations require one puff/administration some others two consecutive puffs/administration. Some of the LAMA/LABA combinations are provided in regimen of two administrations per day (ACL/FF disposable in US and EU, GLY/IND 15,6/27,5 μg disposable only in US, GLY/FF disposable only in US), others in OD formulations (UMEC/VIL and TIO/OLO both disposable in US and EU and GLY/IND 50/110 μg disposable only in EU). In particular It should be noted that the GLY/IND associations in the US is available only at a lower dosage than that used in clinical trials (Vogelmeier et al., 2013; Zhong et al., 2015; Wedzicha et al., 2016) in relation to the differences in the US-approved IND dosage compared to the one approved in the EU.

All these differences in the regimens of administration must be kept in mind and could disorient the less experienced prescribers and the patients switching from one treatment to another.

A separate chapter deals with the treatment of the differences related to the different delivery systems of the different LABA/LAMA combinations. Most of the inhalers for the LABA/LAMA associations are DPI except SMI for Stiolto^®^ and pMDI for Bevespi^®^.

As before discussed each inhaler type has pros and cons that must be considered in the selection of a device for a particular patient. The DPIs, SMI and pMDIs present therefore differences in the size of the particles carrying the drug and therefore in the ability to reach the lung tissue more or less deep. The flow rate able to activate the different inhalers, the need for greater or less coordination of the patient and the preferences of the patients regarding the “taste” of the different formulations are all elements that will play a fundamental role not yet studied in the daily clinical application of these formulations.

## Future Directions

Future studies should indicate clinicians the results of head-to-head comparisons among the different LABA/LAMA associations in order to make less empirical the choice of the combination to be used. Since none of the available LAMA/LABA FDCs have been compared in head-to-head trials, they can only be weighed against each other by indirect comparisons as meta-analysis. Conclusions of that meta-analysis should be interpreted with great caution because of differences in population studied, and differences in primary and co-primary outcomes.

A recent comparison meta-analysis was not conclusive due to the heterogeneity of phase III studies and the few number of studies for some of the investigated drugs (ACL/FF and GLY/FF in particular), but confirmed that dual bronchodilation was always more effective than LABA or LAMA alone, both in lung function and quality of life indices (Calzetta et al., 2016). This meta-analysis suggested that among the different FDCs may exist a gradient of effectiveness, UMEC/VIL slightly improving the effects of GLY/IND (-12 mL), TIO/OLO (-30 mL), and ACL/FF (-49 mL), though statistically significant differences were not observed.

Recently Kerwin et al. (2017) provided data about two cross-over trials comparing efficacy and safety of GLY/IND twice-daily 15.6/27.5 μg versus once-daily UMEC/VIL 62.5/25 μg. Both treatments provided clinically meaningful and comparable bronchodilation. Non-inferiority of GLY/IND to UMEC/VIL could not be declared although between-treatment differences were not clinically relevant (Kerwin et al., 2017).

Kalberg et al. (2016) published data from a 12 weeks’ treatment study with UMEC/VIL 62.5/25 μg that provided similar (non-inferior) improvements in lung function, symptoms, and health status, as well as comparable safety profiles to those achieved with free combinations of a LABA plus LAMA (TIO 18 μg + IND 150 μg).

In order to provide safety and efficacy data to treating physicians, further head-to-head comparative studies applying appropriate non-inferiority limits should be conducted.

### Triple Therapy

It is necessary spending few words on the role of adding ICS to the LABA/LAMA combinations in some particular COPD patient categories. The so-called “Triple therapy” has demonstrated to produce benefits in COPD patients in GOLD group D (Singh et al., 2016) FF 6 μg, GLY bromide 12.5 μg, and beclomethasone 100 μg was compared to FF and beclomethasone in reducing exacerbation frequency providing a -12% of exacerbation frequency. Moreover, TRINITY study compared FF 6 μg, GLY 12.5 μg, and beclomethasone 100 μg to TIO and found that the triple combination therapy resulted in a 20% reduction in the rate of moderate-to-severe COPD exacerbations, improvement in pre-dose FEV_1_ in COPD patients with frequent exacerbations (Vestbo et al., 2017). However, the results of head to head comparison between LAMA/LABA and LAMA/LABA/ICS combinations are still pending. Recently the TRIBUTE study (Papi et al., 2018) compared single-inhaler triple combination of beclometasone, FF, and GLY (87 μg/5 μg/9 μg) 2 inhalations twice per day versus a single-inhaler dual bronchodilator combination of GLY/IND (43 μg/85 μg) one inhalation daily in a group of symptomatic COPD with severe or very severe airflow limitation and at least one moderate or severe exacerbation in the previous year. Authors reported that triple therapy significantly reduced the rate of moderate-to-severe exacerbations compared with GLY/IND, without increasing the risk of pneumonia. Confirming the possible importance of triple treatment in more severe COPD patients. Currently another study is comparing TIO/OLO with ICS/LABA/LAMA triple therapy but results are still waited (Clinical Trials Registry, 2017).

Lipson et al. (2018) recently we compared 52 weeks of a OD combination of FF/UMEC/VIL 100/62.5/25 μg with FF/VIL 100/25 μg and UMEC/VIL 62.5/25 μg. Authors observed that Triple therapy with FF/UMEC/VIL resulted in a lower rate of moderate or severe COPD exacerbations than the other dual combinations in this population. Triple therapy also resulted in a lower rate of hospitalization due to COPD than UMEC/VIL association (Lipson et al., 2018).

Treatment (IMPACT) study will evaluate the efficacy and safety of FF/UMEC/VIL 100/62.5/25 μg versus FF/VIL 100/25 μg or UMEC/VIL 62.5/25 μg, over a 52-week treatment period on COPD D patients. The study was designed to assess not only the value of triple therapy compared to dual therapy, but also the relative comparative advantages of the two dual therapies (ICS/LABA and LABA/LAMA) (Pascoe et al., 2016).

Going ahead, a more personalized approach to therapy is required since we have understood that with the term COPD we define a disease characterized by persistent airways obstruction but at the basis of this has been demonstrated the presence of different phenotypes (Montuschi et al., 2014).

In fact, if we consider asthma COPD overlap syndrome (ACOS), it is clear that identification of these patients will make it possible to get more benefit from the use of ICS compared to patients with other phenotypes. Moreover, the identification of COPD patients with elevated eosinophil count in the sputum may guide the physician in the choice of treatment adding ICS to bronchodilators. These examples allow us to understand the importance of the knowledge of the physio-pathological bases of COPD that will allow in the future identifying treatments more and more tailored beyond the standardized classifications of COPD based on spirometry and frequency of exacerbations.

## Conclusion

LAMA/LABA combinations are recommended in COPD with persistent symptoms or with further exacerbations treated with monotherapy. LAMA/LABA combinations have a synergistic effect rather than just being additive one and have been shown to improve lung function, lung hyperinflation, exercise tolerance, exacerbations frequency and quality of life in COPD. However, a number of questions is still pending and under debate. It is now more and important identifying different phenotypes, so future data are warrant to make clear which combination is more favorable to use, in which patient and at which stadium of the disease, tailoring the treatment to be more and more personalized.

## Author Contributions

MM and AR conceived the idea of the manuscript. AR, VF, FP, and PP contributed to various parts of the text and wrote the manuscript. MN prepared the figures and bibliography. CP, MM, and AR edited the manuscript. All the authors revised and commented on the manuscript.

## Conflict of Interest Statement

The authors declare that the research was conducted in the absence of any commercial or financial relationships that could be construed as a potential conflict of interest. The reviewer BR declared a shared affiliation, with no collaboration, with the authors MN and AR to the handling Editor at the time of review.
